# The Interplay Between Technology Performativity and Health Care Professionals in Hospital Settings: Service Design Approach

**DOI:** 10.2196/23236

**Published:** 2022-01-04

**Authors:** Oluwamayowa Ogundaini, Retha de la Harpe

**Affiliations:** 1 Department of Information Technology Cape Peninsula University of Technology Cape Town South Africa; 2 Graduate Center of Management Faculty of Business Cape Peninsula University of Technology Cape Town South Africa

**Keywords:** agency, health care professionals, technology performativity, sub-Saharan Africa, service design, work activities, mobile phone

## Abstract

**Background:**

The unexpected outbreak of the COVID-19 pandemic and the preventive measures of physical distancing have further necessitated the application of information and communication technologies (ICTs) to enhance the efficiency of work activities in health care. Although the interplay between human agency and technology performativity is critical to the success or failure of ICTs use in routine practice, it is rarely explored when designing health ICTs for hospital settings within the sub-Saharan Africa context.

**Objective:**

The objective of this study is to explore how the service delivery quality is being influenced by the technology-enabled activities of health care professionals at points of care using a service design strategy.

**Methods:**

An interpretivist stance was assumed to understand the socially constructed realities of health care professionals at points of care in a hospital setting. A service design strategy was identified as suitable for engaging health care professionals in co-design sessions to collect data. A purposive sampling technique was used to identify the participants. Open-ended questions were administered to gain insights into the work activities of physicians and nurses at points of care. Qualitative (textual) data were analyzed using thematic analysis. Ethical concerns about the safety and privacy of participants’ data were addressed as per the university ethics review committee and provincial department of health.

**Results:**

The findings show that the attributes of human agency and technology features that drive technology performativity result in an interplay between social concepts and technical features that influence the transformation of human-machine interactions. In addition, the interplay of the double dance of agency model can be divided into 2 successive phases: intermediate and advanced. Intermediate interplay results in the perceived suitability or discomfort of health ICTs as experienced by health care professionals at initial interactions during the execution of work activities. Subsequently, the advanced interplay determines the usefulness and effectiveness of health ICTs in aiding task performance, which ultimately leads to either the satisfaction or dissatisfaction of health care professionals in the completion of their work activities at points of care.

**Conclusions:**

The adopted service design strategy revealed that the interaction moments of the tasks performed by health care professionals during the execution of their work activities at point of care determine the features of health ICTs relevant to work activities. Consequently, the ensuing experience of health care professionals at the completion of their work activities influences the use or discontinuation of health ICTs. Health care professionals consider the value-added benefits from the automation of their work activities to ultimately influence the quality of service delivery. The major knowledge contribution of this study is the awareness drawn to both the intermediate and advanced interplay of human-machine interaction when designing health ICTs.

## Introduction

### Background

This study highlights how the work activities of health care professionals’ shape or are shaped by technology performativity at points of care. The quality of health care services delivered to patients is largely dependent on the ability of health care professionals to productively execute their work activities [1]. Moreover, enabling and contextual conditions influence how health care professionals execute their work activities. Existing literature shows that public health care sectors, particularly in sub-Saharan Africa, are continuously plagued by the prevalent burden of diseases and other recurrent challenges in their health care systems [2]. Some of the challenges are characterized by infrastructural deficiencies, long waiting times, limited accessibility to health care, shortage of skilled personnel, and other human-induced factors [3]. These challenges contribute to some of the contextual conditions that enable or inhibit how health care professionals execute their work activities.

As the number of individuals who require access to health services increases, especially in the public health sector, health care professionals’ work activities require adequate facilitating conditions to perform actions more effectively through the use of relevant tools. The tools relevant to the work activities of health care professionals are paper-based and technology-enabled in the form of health information and communication technologies (ICTs). Health ICTs enable health care professionals to collect, store, and retrieve any health-related information in electronic or digital format to make informed decisions related to diagnosis and treatment. However, when the health ICTs are not adequately designed to align with the work activities and use-case scenarios in hospital settings, they may not adequately serve health care professionals [4]. Therefore, this paper contributes to the literature on how health care professionals perceive the suitability of health ICTs in executing their work activities.

### Challenges Associated With Health Care Work Activities in Sub-Saharan Africa

This paper aims to contribute to how health care professionals can use health ICTs to execute their work activities at points of care in hospital settings. In sub-Saharan Africa, the work activities of health care professionals are usually inhibited by contextual challenges that influence the efficiency of job performance, quality of health care services, and patient satisfaction [5]. These challenges may often result in decision-making errors and a decline in the job performance of health care professionals, which ultimately affects the quality of service delivery. Some of the contextual challenges lead to patients experiencing longer waiting times during visits and, in some instances, a lack of access to health care services [2]. However, to mitigate some of the contextual challenges, health care institutions have invested in health ICTs to enable work activities because of their value-added benefits. These benefits include improved data management, constructive decision-making, improved ease of communication, and enhanced productivity [6].

### Benefits of Technology-Enabled Health Care Work Activities

The goal of introducing ICTs in the health care sector is to enhance the execution of work activities by health care professionals and improve health care service delivery [7]. The use of health ICTs at points of care during and after patient visits helps in improving information administration, collaboration between health care professionals, and in some cases efficient decision-making and diagnosis as well as adherence to treatment. Technology-enabled activities have the potential to positively transform clinical practice and the quality of service delivered by health care professionals.

Depending on the activities being performed, health care professionals can collect, record, and store patient information using hospital information systems both locally and remotely [8]. This enables easier access and retrieval of clinical notes and patient records, including medical history, laboratory tests, and medical images, to assist in decision-making. The use of computers and mobile technologies to facilitate the exchange of information and instant messaging has improved communication and coordination between health care professionals, especially to collaborate or to seek a third specialist’s opinion [9].

Health care professionals rely on software apps and web-based resources to access medical journals and databases [10,11]. These resources enable health care professionals to identify up-to-date medical literature and recommendations that support decision-making regarding drug reference prescription, diagnosis, and at points of care during service delivery. The benefits of health ICTs have resulted in improved efficiencies of manual processes and enabled accountability to manage the frequency of human errors that may occur during the work activities of health care professionals.

Unfortunately, there are isolated instances where health ICTs continue to fail because of suboptimal performance, being unsuitable for clinical-related tasks at points of care, and resistance to change as exhibited by frontline technology users [12]. Other reasons for health ICTs failing postimplementation can be attributed to the lack of adequate consideration for users’ behavioral attributes, particularly agency, during different use cases by the technology designers or hospital management [13].

The technology initiatives of health ICTs in sub-Saharan contexts prioritize primary health and field data collection by community caregivers, patient administration, and billing systems or technology that target special types of diseases such as tuberculosis and HIV monitoring. However, there are only a few studies on how health ICTs enable health care professionals to ensure quality service delivery at points of care in a hospital setting [2]. This paper contributes to the existing literature on how health care professionals perceive the effectiveness of health ICTs during the execution of their work activities at points of care. For this paper, the double dance of agency was used as a lens to identify the theoretical concepts of interplay between health care professionals and technology performativity in a hospital setting.

### The Double Dance of Agency Model

#### Overview

The double dance of agency model incorporates key concepts that show attributes of human agency, the process of agency translation, material performativity, and mediation of contextual conditions [14]. The concepts are strongly rooted within the contexts in which human and nonhuman actors exercise some form of agency and its effects on the intended outcomes of an activity. In this paper, conditions are defined as the circumstances that make certain course of actions more favorable or unfavorable than their alternatives. The root constructs of the conditions include personal histories, social structures, and situational networks. For example, human agency can be influenced by inefficiencies experienced as a result of poor enabling conditions or an overwhelmed system that manages a large population with limited skilled workers. These conditions have a negative effect on the quality of service delivery.

Some of the lived experiences of health care professionals are either enabled or restricted by the elements of social structures such as cultures, rules such as the code of medical practice, strategies, and available resources [15]. However, human actors reserve the willingness to interact in alignment with or against the constructs of these conditions [16]. In the context of health care, professionals use health ICTs based on the presumption of their usefulness and perceived value-added benefits. This is often informed by the desire to improve the inefficiencies of work activities. Subsequently, the properties of human agency and technological capabilities significantly influence the goals and outcomes of work activities during service delivery.

The degree of autonomy associated with agency in machines is influenced by the conditions of human agency in a human-machine network [17]. For instance, a machine does not ordinarily possess the capability to exhibit awareness of self-input and adapt as a reaction to unintended consequences in a context, for instance, a hospital information system. However, it is acknowledged that artificial intelligence (AI) machines have been developed using complex algorithms such as machine learning to offer a relative degree of autonomy to machines [18]. In AI systems, the machine is aware; it can interpret and continuously adapt to a change in the conditions of the human environment, for example recommender systems. In this study, the hospital information systems and mobile apps were not built with advanced AI. The health ICTs in this study were legacy machines that required the input, negotiation, and transformation of human efforts into desired outcomes.

#### Human Agency

Agency is defined by Giddens [19] as the “capability to make a difference,” whereas in the social cognitive theory it is defined as “choice to act intentionally” [20]. At face value, these definitions extend to human actors and machines; however, the degree of agency is influenced by conditions such as personal history, social structure, and situational networks [14]. For instance, human agency is characterized by attributes of self-awareness, context-awareness, and intentionality [21]. These attributes enable humans the autonomy and choice to make conscious decisions that influence the outcome of their activities. Technology is designed by humans as part of the solutions informed by their adverse lived experiences, expectations, intended outcomes, and their desire to exhibit power. Therefore, the authors argue that in health care, agency is ascribed mainly to humans in a human-machine interaction, whereas technology is an enabling tool to efficiently facilitate interactions of humans when performing tasks.

Users have sometimes made efforts to accept and adapt to how ICTs are designed to work for meeting their needs and expectations or interests. A study on the Internet of Things (IoT) [22] investigated the balance between human agency and object agency. This study explored the perceptions and attitudes of IoT users. The findings showed that users expressed dissatisfaction with the limited ability to exercise their agency, suggesting that future designs of IoT technologies should be aligned with the affordances and habitual needs of its end users and at the same time guarantee more control to humans. Technology designers could inadvertently focus on the capabilities of a technology innovation for a particular purpose without extensive consideration of the user agency and the context of use. Therefore, technologies might yield results anticipated by the designer but not necessarily informed by the users’ requirements.

#### Technology Performativity (Machine Agency)

Unlike human actors, machines do not have a degree of agency to act independently [14]. A machine translates its input instructions as instructed by human actors to yield a defined output or outcome, which is referred as material or technology performativity [23]. For instance, a machine does not possess the capability to exhibit awareness of self-reflection and adjustment in reaction to the unintended consequences in a pre-established context. Typically, the capability of health ICTs is attributed to their features [24]. These features may include, but not restricted to, the screen size, type of keypad, computing power or processor, amount of storage memory, wired or wireless-enabled technologies, sensors, in-built cameras, and hands-free functions [25,26].

In the context of health care, professionals use health ICTs such as hospital information systems and mobile apps, which are perceived as useful in improving the inadequacies of paper-based and manual systems [10]. This perceived usefulness influences the routine use of health ICTs, as informed by their value-added benefits [27]. Conversely, the need for humans to discard or adapt to an implemented technology often arises from scenarios in which a technology does not adequately serve the intended purposes of work activities in a timely manner [28]. Thus, claims of machine agency [29] in a sociotechnical network within the health care context leaves more questions to be addressed than answers.

In this paper, authors draw from existing literature on the concepts of human agency, the perceived machine agency, and the interplay between these 2 concepts to understand the effect of health ICTs on the work activities of health care professionals in a public tertiary hospital setting.

## Methods

### Service Design Approach

The authors adopted a service thinking approach to particularly understand how the work activities executed by health care professionals in a hospital setting are enabled or inhibited by health ICTs during service delivery. The research was qualitative in nature and adopted a service design strategy to collect the desired information from a sample of health care professionals within the research population of a hospital. The service design double diamond model was adopted as a strategy because it enables human-centered collaboration that visualizes human interactions along a timeline and the values-in-use of a service [30]. The authors engaged with health care professionals to understand how they executed their work activities and to identify how they used health ICTs. The strength of this strategy was that the authors could compare the similarities or differences between *what participants say* and *what they actually do* through co-design sessions. In this study, the application of service design was adopted to engage with participants and obtain a holistic understanding of how work activities are currently executed by health care professionals and does not seek to change the established medical practice workflow at the points of care. The service design double diamond model provided a human-centric approach to acquire primary data on the work activities executed by health care professionals.

The service design double diamond model is broadly categorized into 4 phases. In this study, the authors adopted the first 3 phases to collect data from the participants. In the first phase, the authors engaged with secondary data or existing literature to identify how health ICTs have been used by health care professionals at points of care in hospital settings. This prompted the objective of this study. In the second phase, the researchers used open-ended questions to define the touch points of health care professionals’ work activities. Touch points are instances where human actors and objects interact [31]. The touch points enabled the authors to break down the interaction moments of the activities performed by health care professionals during health care service delivery at points of care.

In this paper, touch points are the cluster of interaction moments where health care professionals perform tasks during the execution of their work activities to deliver services. In the third phase, the researchers identified the features relevant to the design of health ICTs, as informed by interaction moments identified in the previous phase. Only 3 phases were applied in a cross-sectional approach, as the fourth phase required a longitudinal investigation to deploy and evaluate a physical artifact, which would have been dependent on permissions from the provincial department of health.

### Recruitment

#### Clinical Setting Context

Hospital H is an academic hospital in the Western Cape province of South Africa that provides specialized health care services, trains higher education institution medical students and promotes research. As of 2016, hospital H catered to >3.4 million people in the geographic area where it is situated, of which over 599,885 patients visited the hospital per year. The hospital is notable for its progress in the implementation of eHealth information systems, such as hospital information systems and mobile health (mHealth) apps.

#### Data Collection Process

Before engaging with the participants, we applied for an ethical clearance to collect data from the university ethics research committee before approaching the Western Cape Provincial Department of Health. The researcher (OO) was contacted by the manager of hospital H after deliberations by the provincial and the hospital’s ethics committee, who indicated that ethical concerns had been mentioned and addressed. After ethical clearance was granted, emails were sent to the heads of clinical departments of hospital H, to explain the purpose of the research and obtain buy-ins from the top management of the hospital.

Purposive sampling was used to identify the participants. The sample size was selected from the clinical units that provided medical imaging and reporting. The rationale for this sample selection was that the doctors and nurses involved in medical imaging and reporting used different technologies to execute their work activities that involved text and image diagnosis, treatment, and reporting. Informed consent was signed by the participants, indicating that they understood the implications of participating or withdrawing their participation willingly from the research and that their details would be kept confidential when reporting the findings.

Other key stakeholders in the health sector, such as patients, hospital managers, vendors of health ICTs, and policy makers, were not involved in the service design process because this study specifically focused on how the types of health ICTs used by health care professionals were perceived to shape their work activities at points of care during service delivery.

The health care professionals included 4 nursing staff, 6 orthopedics, and 2 ophthalmologists, as described in the profile table (Table 1). The initial sample size was 20, but health care professionals are one of the most difficult research populations to contact, arguably because of their busy schedules [32]. The saturation point was guaranteed because the research focus was on work activities and not on individual behaviors or attitudes. Furthermore, the work activities were similar for each group of health care professionals, which guaranteed the likelihood of no new additional information [33].

**Table 1:** Profile of health care professionals in hospital H^a^.

Participant ID	Area of specialty	Estimated years of practice	Method of engagement
RH_1	Ophthalmology registrar	6	Interview
RH_2	Ophthalmology registrar	8	Interview
RH_3	Orthopedic consultant	10	Interview and co-design
RH_4	Orthopedic registrar	7	Interview and co-design
RH_5	Orthopedic registrar	1	Interview
RH_6	Orthopedic registrar	3	Interview
RH_7	Orthopedic registrar	4	Interview
RH_8	Orthopedic registrar	5	Interview
RH_9	Deputy nursing manager	32	Interview
RH_10	Nursing area manager, theater	25	Interview and co-design
RH_11	Nursing area manager, intensive care unit	20	Interview and co-design
RH_12	Nursing area manager, trauma	25	Interview and co-design

^a^The respondents have been addressed here using pseudonyms starting with RH_, where R denotes respondent and H denotes hospital.

#### Co-design With Open-ended Questions

The co-design activities were performed in a comfortable location within the hospital as selected by the participants; subsequently, the research objective was explained to the participants. The facilitator (OO) provided cut-out graphic representations of the doctors, nurses, and tools used for work activities to the participants, as observed in the literature. Then, large sheets of paper, pencils, erasers, and stickers were provided as writing materials for the participants to illustrate their workflow. According to Debrah et al [34], the cut-out probes of actors and tools provide an opportunity for participants to visually express their actions and make sense of the drawbacks of their work activities.

The facilitator asked the participants to visually illustrate how they performed their tasks and the different tools they used. Participants used the paper cut-outs to represent themselves as actors on a large piece of paper using stickers and provided practical descriptions of their actions at points of care. The outcome of the first task was a visual illustration of the user journey maps of health care professionals from the first encounter with a patient until they were discharged or deceased (Multimedia Appendix 1).

Subsequently, the researcher used the visual illustrations to identify the touch points within the service delivery process. The facilitator then inquired about any challenges experienced by health care professionals using open-ended questions. Participants described their work activity challenges and the resulting effects attributed to the use of health ICTs. The discussion between the facilitator and participants hinted at how health ICTs could be best suited for their work activities. The outcome of the discussion summarized the characteristics of the expected features of a fit-for-purpose technology as shown in the summary of co-design analysis from engagement with physicians and nurses (Table 2).

**Table 2:** Summary of co-design analysis.

Themes and touch points of work activities		Interaction moments of touch points	Tools
**Illustration of work activities**			
	Patient consultation	Verbal communication	Voice
	Patient consultation	Notes writing	Pen and paper
	Patient consultation	Referrals and communication	Smartphone
	Booking and retrieval of clinical examinations	Requests of laboratory tests and imaging using a screen and keyboard	Desktop computer
	Booking and retrieval of clinical examinations	Access and retrieval of tests and image results using a screen and keyboard	Desktop computer
	Nursing administration of patient care	Triage patient admission, transfer, or discharge	Voice; pen and paper
	Nursing administration of patient care	Report writing	Voice; pen and paper
	Nursing administration of patient care	Communication with other professionals	Voice; pen and paper
**Challenges of work activities**			
	During patient consultation	Notes writing; delayed access to digitized paper record	Pen and paper; desktop computer
	Referrals	Uncontrolled interruption	Smartphones
	Nursing administration of patient care	Cumbersome report writing	Pen and paper
**Features for ideal technology-enabled work activities**			
	Remote consultation	Record verbal communication	Mobile device with readable screen size and voice recorder or voice recognition
	Remote consultation	Write or update feature	Mobile device with readable screen size and voice recorder or voice recognition
	Nursing administration of patient care	Report writing	Tablet or smartphone with touchscreen and a preloaded database
	Nursing administration of patient care	Triage patient admission, transfer, or discharge	Tablet or smartphone with touchscreen and a preloaded database

#### Data Analysis Process

In this study, the findings were dependent on the operationalization and frequency of attribute occurrences that defined the key concepts of the research objective. The co-design sessions with the participants were recorded and transcribed from audio to verbatim text. Each co-design activity lasted for 60 minutes. We analyzed the collected data using the thematic analysis technique to identify the attributes that implied words or phrases in the research objective. This technique was guided by conceptualization and operationalization.

Conceptualization process involved identifying and defining key concepts embedded within the phenomenon being investigated. To further simplify the analysis process, the attributes that determined or quantified each key concept were identified from the data transcripts; this is known as operationalization.

The words or phrases identified were assigned descriptive codes in a process known as open coding. In this study, open coding was applied to the transcribed qualitative data to identify and categorize how service delivery was being shaped by technology-enabled activities of health care professionals at points of care (Multimedia Appendix 2). Hence, coding was performed in several iterations to exhaustively sort the data according to the hermeneutics circle prescribed for interpretive studies [35].

## Results

### Overview

The outcome of co-design with the participants showed that health care professionals used technology in the form of mobile devices and desktop computers and were aware of technology features relevant to their work activities at points of care.

The data analysis process showed that work activities are characterized by patient care and information administration, diagnosis, and treatment to improve the state of the patient’s well-being. Therefore, the authors sought to establish the tasks performed by health care professionals, the technologies used to execute work activities, and the expected outcomes of specified technology-enabled work activities.

### Background to a Health Care Professional’s Work Activities

The nature of health care professionals’ work activities requires mobility—from wards to clinics to theaters within the hospital. When asked to describe their daily work activities, the doctors mentioned patient consultations at clinics or wards and clinical procedures on patients (respondents RH_1, RH_2, RH_3, RH_4, and RH_5). One participant responded as follows:

A typical day for us at the moment will be one of two things, either a theatre day where we would go to theatre after our morning discussion, operate the patients and after the case is in theatre we do a ward round. We will have our morning meetings...after, we go to the clinic where we see our patients after which we would also do a ward round of the patients we have in-hospital and obviously we have the days that we are on call for the hospital.

RH_4

These activities require administrative tasks, particularly the documentation of patient information that are newly generated or modified. For instance, it was necessary for a health care professional to have quick access to patients’ records in cases of emergency, scheduled visits, and unscheduled follow-up visits:

The nurse does the checklist...checks the patient’s files. Now, she checks for the x-rays and all the necessary docs. For example, the consent form. During the surgery she (the nurse) writes down the complete recordings in the theatre book before taking it to the data capture or scan centre.

RH_10

The responses indicated that the clinical and administrative functions of health care professionals require information administration enabled by essential tools. These essential tools were paper-based methods and health ICTs to facilitate the collection, storage, update, retrieval, and exchange of patient information locally or remotely to aid decision-making and provide quality health care services.

### Purpose of Health ICTs During Work Activities at Points of Care

Health care professionals indicated the use of hospital information systems to manage patients’ records and facilitate booking requests. For instance, physicians perform remote consultations and communications that require the exchange of information with colleagues from other health care institutions. Health care professionals described the purposes for which health ICTs have assisted in automating their work activities. The health ICTs described included the iSite (Philips) picture archiving and communication system (PACS), enterprise content management (ECM; Oracle Corporation) system, nursing information management system (a computerized procurement system developed by the Western Cape Provincial Department of Health, South Africa), and the VULA mobile app (developed by Dr William Mapham, a South African ophthalmologist).

When asked to mention the ICTs used to support their work activities, the doctors described the VULA app as a mobile app used to facilitate referral management. One physician described VULA as follows:

smartphone based app where doctors and health care professionals, that includes more than just the doctors, can have direct communication with the on-call doctor or Orthopedics person, to ask for advice or refer patients to us. That’s by a means of a list of questions that we ask or that’s asked on the app and photos of X-rays that can be sent through to us.

RH_4

The VULA app was described as a mobile app designed to enable health care professionals consult with each other and facilitate communication in the form of exchanging patient history, asking or receiving medical advice and sending clinical images, and making informed decisions remotely. It is used to manage trauma referrals from private and other public peripheral hospitals to tertiary hospitals:

In our clinics, for all patients that are seen notes are made by hand and those notes as well as all referrals goes into a patient’s folder. All those notes are sent to the scan department, get scanned into our ECM and eventually do become available on a computer.

RH_3

The ECM and PACS are systems mostly used by physicians to access patient records and to request or retrieve medical and pathology test results from the clinical laboratory. Participants from the nursing department confirmed that a technology tool was used to support administrative activities involved in the admission, care, discharge, or transfer of patients. Nurses referred to the technology as a nursing information management system. One of the nurses explained as follows:

The ICT tools are used by the nurses to register patients as they are being admitted; to transfer patients; to take patients off the system if they are discharged or die in the hospital; we use it to order food and we use it to order stock in the hospital environment.

RH_9

Health care professionals mostly recounted the value-added benefits associated with the use of health ICTs for their work activities regarding electronic documentation of patient records, access and availability, referrals, communication, and consultation.

### Strengths in Technology-Enabled Work Activities of Health Care Professionals

The evidence of a transformed work process is usually evident in the value-added benefits attributed to the use of technology and its outcomes because health ICTs are designed to improve on the inefficiencies of work activities and enable quality health care services. The study findings show that health care professionals experience easier and quicker access to the patients’ information by using ICTs. When asked how the ECM system assists work-related activities, one of the doctors responded as follows:

It helps to quickly access the patient’s folders and previous notes, previous history of the patient and it speeds up especially when you see the patient on follow up.

RH_1

Clearly, access to patient information at points of care is important to health care professionals’ work activities. However, this information must be available in an electronic format in a timely manner. Regarding the PACS and the VULA mobile app, another participant mentioned the following:

Before PACS and VULA app, we had the hardcopy X-rays. And they would get lost or misplaced somewhere or you would want to discuss a case here and then the X-rays would be in the ward. So just to have the PACS on any computer, you would have the X-rays available and then do your planning and everything as well. And you could draw your lines or do your templating on the PACS itself...You can’t lose the X-rays.

RH_3

The responses imply that health ICTs have transformed the work activities of health care professionals in tertiary health care settings, based on their contribution to the effectiveness of performing tasks and time efficiency. For instance, clinical notes that were handwritten on paper, hard copy X-ray images, and other medical scans are being documented electronically on the ECM and the PACS. The patients’ records are readily accessible and retrievable digitally using desktop computers or mobile devices, regardless of the location of a health care professional within the hospital. Physicians RH_2 and RH_3 expressed that the management of patient referrals had improved as the process had simplified and the number of unnecessary referrals had reduced. One physician stated that the mobile app helped as follows:

Lessens trauma burden on our emergency department and Orthopedics. Because we saw that lot of times, people were referred here that didn’t need to be referred here; that could be managed at a primary or secondary level hospital. I think we have achieved that, to try and limit unnecessary referrals...That has helped a lot.

RH_3

The use of mobile devices such as smartphones and software apps such as the VULA app enables physicians to manage referrals and reduce unnecessary visits to the hospital, thereby reducing the number of patients seen at outpatient clinics. This saves time and cost implications associated with mobility for patients and health care professionals, especially in terms of consultation and communication. Health care professionals can communicate directly, view necessary medical images remotely, and save time on patients’ diagnosis without having to arrange for scheduled visits and physical contact.

### Limitations in Technology-Enabled Work Activities of Health Care Professionals

The adoption and the continued use of health ICTs are often regarded as complex exercises, especially given the peculiarities of contextual and infrastructural conditions such as the number of patients attended to by the health care professionals, interruptions in health ICTs, and internet connectivity. Subsequently, these factors result in challenges and may inhibit the execution of technology-enabled work activities. The research findings indicate that paper inadequacies, technology downtime, and time inefficiencies are the main challenges experienced by health care professionals. For instance, one of the health care professionals explained that the VULA mobile app negatively disrupts patient consultation:

During the day it actually interferes and it slows you down massively. Definitely, because you have a lot of patients that you need to see, you need to answer the phone at ER, you need to answer your bleeps and then you also get VULA referrals.

RH_1

Health care professionals also expressed concerns that attending to their phones during physical consultations might be perceived as unprofessional by patients. Similarly, another physician mentioned the following:

The biggest challenge with VULA app is to be able to find time during patient consultations to also answer to referral doctor’s questions; it takes a lot of multitasking. And when disrupted by calls and VULA referrals, it takes much longer to complete a consultation with a patient.

RH_2

Despite the benefits of the VULA mobile app to enable teleconsultation and facilitate communication between health care professionals, there were claims that it disrupted work activities and extended the time taken to complete these activities. A participant expressed a preference for paper-based tools to perform tasks over ICTs because of their impact on job performance in case of imminent failures:

I prefer paper based for note taking but IT based for outside referrals. The problem with technology is that when there is a problem with it and we need to revert to paper based work it causes issues and delays; it’s all good until the ICTs fails.

RH_5

The PACS or ECM downtime impedes workflow in the sense that health care professionals are unable to perform tasks that largely depend on the use of electronic systems. Subsequently, the doctors would revert to a paper-based system to facilitate the progress of their work activities at points of care:

When the ECM is down then it’s a big problem because then you basically can’t go on with your work. You can’t book a patient for any surgery. If you haven’t screened your patients yet; you need to access that information...but then you have to see the patient again. Then it’s basically the same as falling back onto paper system

RH_1

With regards to the PAC system, the negative part to that is not all computers always work. I think that’s the biggest challenge, is when electronics don’t work. Then it’s a massive irritation, if we can’t see X-rays and you’ve got a clinic full with 40 patients.

RH_4

The responses reflect the contextual conditions of multitasking and the number of patients that need the attention of health care professionals at points of care. Regarding infrastructure, the participants suggested a shortage in desktop computers and unreliability in the ones available. Unfortunately, the mobile app being used to manage referrals and facilitate the exchange of patient information can be disruptive and time consuming during the execution of work activities.

The analysis of the findings indicates that technology-enabled work activities executed by health care professionals are influenced by contextual factors and the extent to which health ICTs fit the tasks being performed at points of care. Furthermore, the health ICTs being used at the points of care are associated with unintended consequences that inhibit the work activities of health care professionals during service delivery. Despite the ensuing inhibitions caused by the unintended consequences experienced during technology-enabled activities of health care professionals, the findings show that there is a continued use of the health ICTs. The findings have been interpreted in the *Discussion* section to clarify the essence of these analyses.

## Discussion

### Principal Findings

In health care settings, information is generated by health care professionals at every point of care; hence, there is a need to consolidate the information trail. Before the introduction of technology in hospital settings, the process of managing information required ample use of paper to keep records [36].

The search for paper-based records takes a lot of time, thereby increasing patient waiting times; paper folders are susceptible to being easily misplaced when transferred to and from clinics or between health care professionals [37]. These are some of the challenges experienced by health care professionals during their work activities at points of care. However, the introduction of health ICTs has automated many tasks, making it easier, faster, and safer for health care professionals to execute their work activities.

Therefore, the analysis of the findings is categorized using the concepts from the double dance of agency model to discuss the effects of technology performativity on the work activities of health care professionals and their unintended consequences on the interplay between the attributes of human agency and technology performativity.

### Effects of Technology Performativity on the Work Activities of Health Care Professionals

The findings show that human agency, lived experiences, contextual conditions, and needs are the underlying drivers of technology performativity. Health ICTs enhance efficiencies associated with tasks relating to consultations, referrals, and treatment plans for health care professionals at points of care. In particular, mHealth ICTs offer a platform for health care professionals to communicate, retrieve, and exchange information in a context where physical distancing is required in order to reduce the spread rate of COVID-19 within the African context.

An improved quality of health care service delivered by doctors and nurses is the desired goal and anticipated outcome of the decision-making process at points of care [1]. Despite the infrastructural glitches, health care professionals expressed their lived experiences on the impacts of health ICTs on their tasks during work activities. Doctors continue to make use of health ICTs based on the presumptions and experiences of their suitability and capabilities in improving the inefficiencies in their work activities in a timely manner. This is often informed by a comparative measure of the past experiences of health care professionals while performing tasks and a desire to improve the efficiency of their work activities. For example, duplication of paper records and the susceptibility of hard copy records to loss or damage from incessant handling are reduced significantly because of the use of health ICTs. This relates to the studies conducted by Bervell et al [37].

In this study, the authors established that although the intent of technology-enabled work activities is to improve efficiency, the suitability of health ICTs to perform tasks and technology features determines the quality of service delivery outcomes. One of the themes identified from the data analysis is unintended consequences, which have an underlying effect on the efficiency of how hospital information systems facilitate the retrieval of digitized records by doctors. Another example is the reported interruptions during face-to-face consultations between physicians and patients from trauma referral notifications on the physicians’ VULA mobile app.

### Unintended Consequences of Technology Performativity on Technology-Enabled Work Activities

The properties of human agency and technology performativity jointly influence the outcomes of work activities [13]. When there is a lack of synergy or overdependence between the attributes of human agency and technology features, it results in unintended consequences. These consequences are more like surprises or occurrences of unplanned outcomes other than desired intentions [38]. The findings indicate that there are differences between the use of health ICTs and the nature of work activities executed by health care professionals. Being conscious and self-aware of the timeous nature of care service provision, health care professionals tend to experience discomfort when health ICTs interrupt the timely completion of their work activities. According to Adeleke et al [39], time constraint is a major inhibiting factor in the use of health ICTs by health care professionals at the points of care. Despite the perceived suitability of the VULA mobile app and the ECM system in reducing unnecessary referrals and facilitating quicker access to patient records, respectively, sometimes these tools increase the workload of doctors and delay the completion of work activities at points of care.

Mobile devices and health apps can facilitate consultation and communication between health care professionals. However, health care professionals felt it was improper to constantly stare at their phones to input a function or to receive an output, such as accessing or retrieving records, while attending to patients. In this case, it is evident that the design functionalities of mHealth ICTs influence how health care professionals execute their work activities. The participants expressed concern about how their patients would interpret their constant interaction with the mobile device. According to Yahya et al [40], some health care professionals are concerned that the use of mHealth ICTs might be misconstrued by their patients during consultations at points of care.

In this research, it is argued that self-awareness is a key mediator that can influence how health care professionals perceive the suitability or discomfort attributed to the use of health ICTs. However, this finding contrasts with the study by Kabanda et al [32], where the authors found that participants were comfortable using their mobile devices during consultations. The inconsistency of results within the same research context further clarifies that the interplay between human agency and technology performativity can be experienced differently and is not generalizable across clinical settings, especially because of the effects of choice and self-awareness.

Technology abuse is another unintended consequence that may be experienced by health care professionals. In addition, the increased workload of multiple referrals during patient consultation could be attributed to how the VULA mobile app was designed, that is, without adequate consideration of its impact on patient-physician interaction at the points of care. For example, one of the participants stated that “most people are quite negative about it because it gets abused.” The response indicated that the use of the VULA mobile app drew negative feelings because there were little to no restrictions on the extent of its use by referring doctors “but no one really complains*.*” It is evident that, after a period of time, health care professionals eventually become used to a system that is fit-for-purpose in the course of their work activities. This aligns with the longitudinal study conducted by Vaghefi et al [24] on the continued use of mHealth technologies. Subsequently, health care professionals pay less attention to the few challenges and ultimately make a choice to either adapt and be satisfied or be dissatisfied and eventually discard the implemented health ICTs.

The manner in which health ICTs are used by health care professionals to enable the interaction moments of tasks at points of care is influenced by the capabilities of tools to enhance the productivity of work activities and the knowledge of health care professionals. However, the benefits of health ICTs, including mHealth technologies, to enable communication, retrieval, and exchange of information during work activities of health care professionals may be partially realized, as indicated by Martin et al [41].

Workarounds are examples of desired unintended consequences. Workarounds can be defined as the use of technology other than its intended use [42]. In this paper, a workaround is described as the use of an alternative means to efficiently perform certain tasks effectively and achieving the intended outcome in situations where the initial means of action are perceived as inappropriate. Workarounds establish that human agency attributes ultimately influence perceived technology performativity in the event that a technology is unavailable or is associated with usability challenges.

The authors conclude that during technology-enabled work activities, health care professionals can use tools such as mHealth technologies to perform and coordinate their work actions, particularly to address the issues of timeliness associated with location constraints. The interplay between attributes of human agency and technology performativity influences the acceptance or nonacceptance and use and ultimately transforms the purpose of the work activities into intended outcomes [43]. On the basis of the continued use of health ICTs in clinical settings of hospital H despite challenges experienced by health care professionals, the authors have concluded that there are levels to the interplay between human agency and technology performativity.

### Levels to the Interplay Between Human Agency and Technology Performativity

Despite the unintended consequences experienced by health care professionals, they continued using mHealth apps or devices and hospital information systems for their work activities. For this study, the interplay explained by Rose and Jones [14] is further broken down into levels that are characterized as intermediate interplay and advanced interplay. These 2 levels are determined by the interaction between humans and machines and are the reasons because of which health care professionals continue to use health ICTs for technology-enabled work activities at points of care.

At the intermediate interplay level, health care professionals use health ICTs to enable the tasks of their work activities based on the capabilities of its features at initial interaction. The lived experience of the initial interactions results in a perception of the suitability or discomfort of the technology during work activities [44]. Here, it is argued that health ICTs are fit-for-purpose but not particularly useful because of the contextual conditions of use. Ultimately, health care professionals are likely to discard health ICTs or adopt an alternative technology that is not specifically designed for health-related tasks such as instant messaging through social media apps or revert to using paper.

In the advanced interplay level, health care professionals eventually adapt despite the contextual conditions of use. In this study, health care professionals tended to become more familiar with the use of health ICTs, largely because of their many benefits in contrast to the challenges experienced during and after work activities. Hence, it can be inferred that the continued use of health ICTs indicates that health care professionals are able to negotiate and become satisfied or dissatisfied. According to Cresswell et al [45], sociotechnical systems become complex over time and hence, it is important to understand how technology is eventually normalized by social actors in their context of use. Hence, the benefits of using health ICTs may outweigh the issues that inhibit their seamless use by health care professionals or the system may ultimately be discarded [46]. In these 2 levels of interplay presented by the authors, the lived experiences and cost–benefit analysis of health care professionals during technology-enabled work activities determine the eventual acceptance or nonacceptance of health ICTs.

The authors acknowledge that both humans and technology can make a difference in human-machine interactions. However, the types of health ICTs identified and used in the context of this research do not act intentionally. The technology is designed by humans as part of a solution informed by historical events of adverse lived experiences, perceived expectations, desired outcomes, and the need for humans to exhibit some level of power. Hence, we argue that in health care, agency is ascribed mainly to humans in a human-machine interaction, and technology is viewed as an enabling tool to facilitate the actions of humans or in the interaction moments of tasks.

This study established that the outcome of work activities is largely dependent on how the attributes of human actors necessary to perform the tasks are interleaved with the perceived capabilities of the means of action. In other words, the interplay between health care professionals and implemented health ICTs is influenced by intentionality, self-awareness, medical practice, and the capabilities of the technology features.

### Limitations and Future Research

The limitation of this qualitative study is that it is subjective and context-based to a tertiary hospital in Cape Town, South Africa. Hence, the findings are not generalizable, but could be relevant for environments with the same contextual factors as Western Cape province, South Africa. The service design approach enabled the strategy used for data collection; however, its fourth phase needs to be executed to contribute toward the richness of the approach. In the future designs of health ICTs by user experience and interaction designers for the sub-Saharan context, factors such as adaptability, workarounds, and infusion should be extensively addressed before the development phase. Other stakeholders such as policy makers, business analysts, and even health care professionals need to periodically evaluate the existing health ICTs to identify the unintended consequences of technology-enabled work activities at points of care to prevent an abrupt discard of the technologies.

### Conclusions and Contribution

In this study, human agency and contextual conditions are the underlying driving agents for technology performativity. Service design contributed toward making a sense of the findings that health ICTs address the perceived inefficiencies of work activities. Although a change cannot be influenced by the current flow of work activities within the clinical settings, the tasks performed at points of care shape how health ICTs could be designed and improve the lived experiences of health care professionals at points of care during the overall service delivery process. Therefore, the interplay highlighted by Rose and Jones [14] should be expanded to include an intermediate and advanced interplay between human agency and technology performativity.

Designers and implementers of health ICTs need to take into account the information-intensive nature of health care settings; hence, a networked desktop or mobile system that easily facilitates read, write, search, and edit actions on patient records would ensure up-to-date health information at all times. An updated electronic record available to doctors and nurses would improve decision-making and the accuracy of diagnosis during health care service delivery. Thus, this paper contributes to claims on the cost–benefit analysis of automation and the synergy between human agency and technology performativity in health care contexts.

## Appendix

Multimedia Appendix 1 Sample of user journeys generated from co-design sessions with physicians and nurses.

Multimedia Appendix 2 Analysis and coding process of co-design transcripts.

## Abbreviations

AI: artificial intelligence
ECM: enterprise content management
ICT: information and communication technology
IoT: Internet of Things
mHealth: mobile health
PACS: picture archiving and communication system
