# Supplementary material for: The Interplay Between Technology Performativity and Health Care Professionals in Hospital Settings: Service Design Approach
Source: JMIR Form Res. 2022 Jan 4;6(1):e23236. doi: 10.2196/23236 (PMC8767474; doi:10.2196/23236)
Supplement: Multimedia Appendix 1 [file formative_v6i1e23236_app1.pdf]

Sample snapshots of user (healthcare professionals) journeys during technology-enabled work activities in hospital H

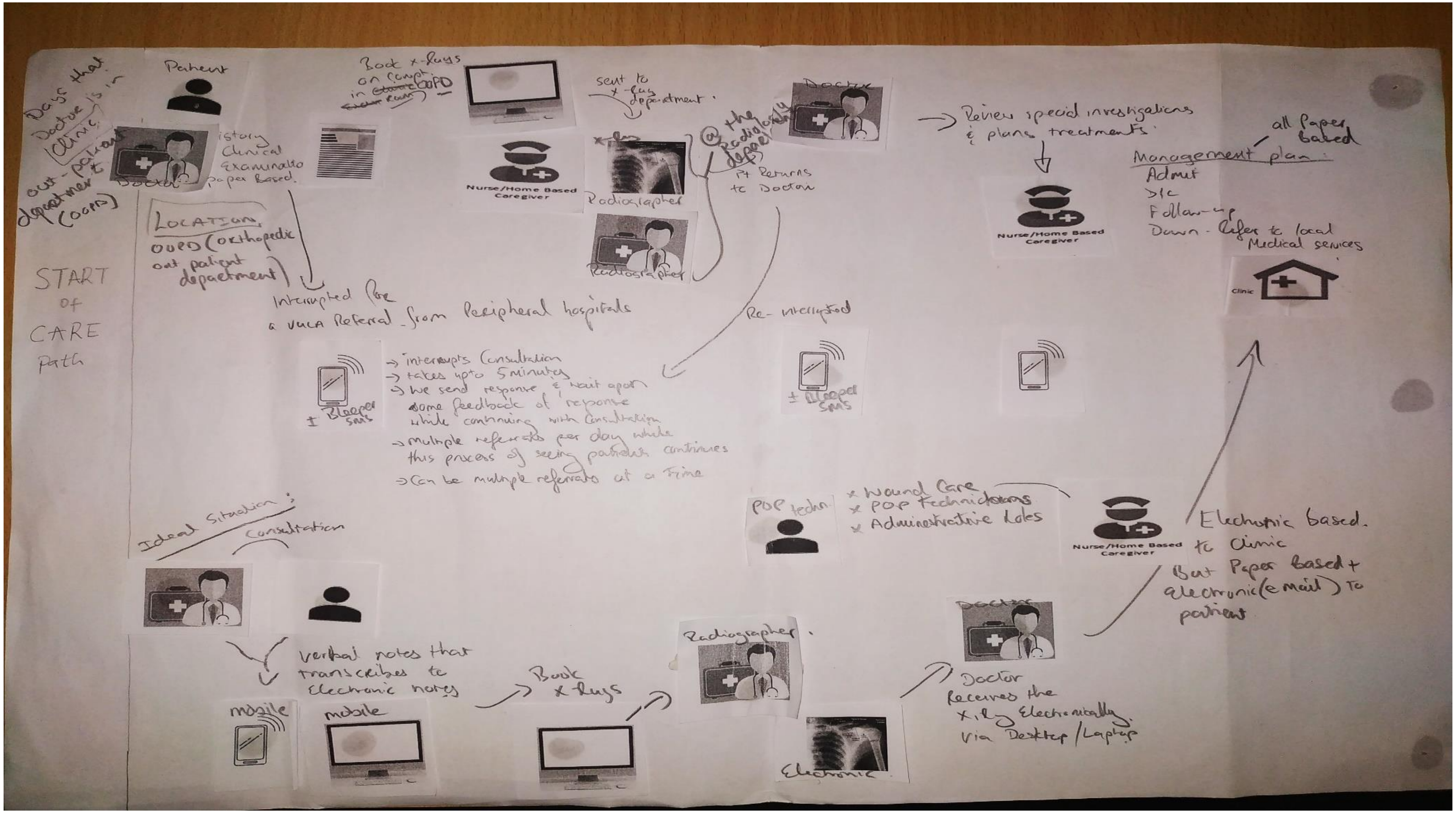

Figure 1: visual illustration of doctors in hospital H

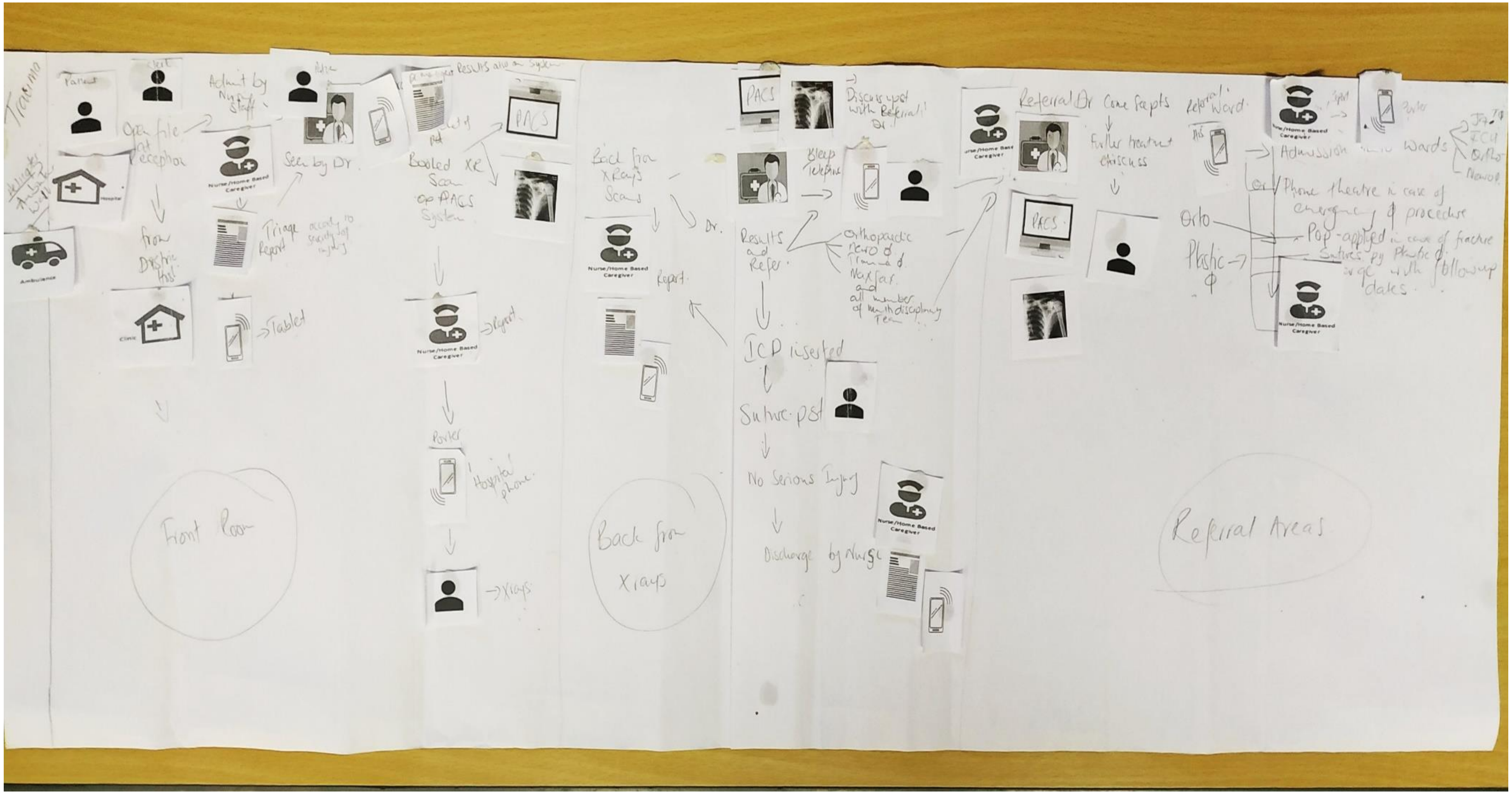

Figure 2: visual illustration of nurses in trauma wards of hospital H

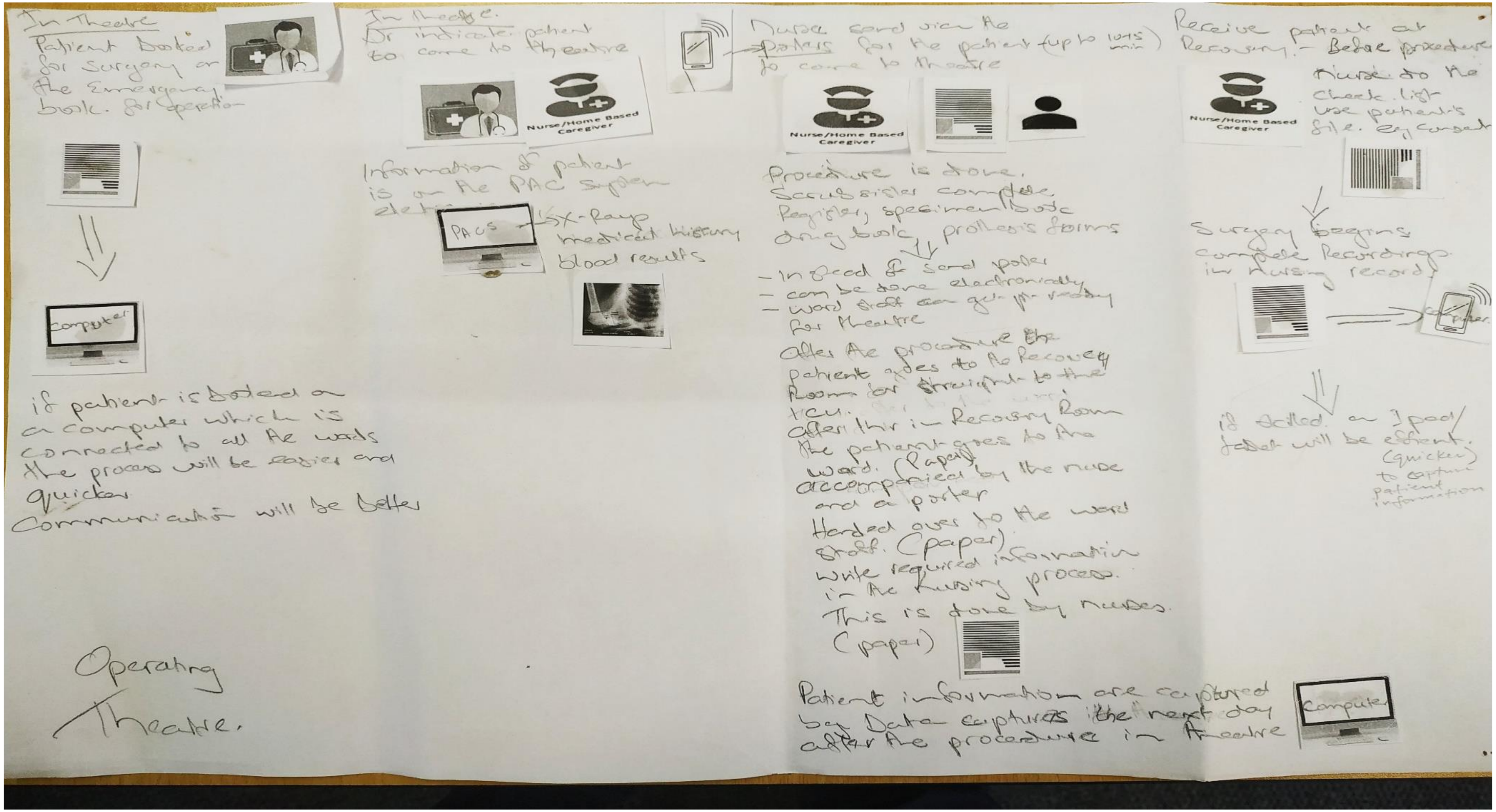

Figure 3: visual illustration of nurses in the theatre units of hospital H
