# Supplementary material for: The Interplay Between Technology Performativity and Health Care Professionals in Hospital Settings: Service Design Approach
Source: JMIR Form Res. 2022 Jan 4;6(1):e23236. doi: 10.2196/23236 (PMC8767474; doi:10.2196/23236)
Supplement: Multimedia Appendix 2 [file formative_v6i1e23236_app2.pdf]

APPENDIX 2: Thematic analysis coding of co-design with open-ended questions.

DESCRIPTIVE CODES	CATEGORIES	sub-THEMES	THEMES
morning handover meeting	administrative duties and planning	ADMINISTRATIVE DUTIES	WORK ACTIVITY OF HEALTHCARE PROFESSIONALS
prepare theatre list for call or extra trauma lists	administrative duties and planning	ADMINISTRATIVE DUTIES	WORK ACTIVITY OF HEALTHCARE PROFESSIONALS
academic meetings (and presentations)	academic engagements	ADMINISTRATIVE DUTIES	WORK ACTIVITY OF HEALTHCARE PROFESSIONALS
operate the patients	perform clinical procedure on patients	CLINICAL DUTIES	WORK ACTIVITY OF HEALTHCARE PROFESSIONALS
trauma theatre	perform clinical procedure on patients	CLINICAL DUTIES	WORK ACTIVITY OF HEALTHCARE PROFESSIONALS
ophthalmology clinic	consultation with outpatients in clinic	PATIENT CONSULTATION	WORK ACTIVITY OF HEALTHCARE PROFESSIONALS
Orthopaedic clinic	consultation with outpatients in clinic	PATIENT CONSULTATION	WORK ACTIVITY OF HEALTHCARE PROFESSIONALS
consulting patients in the outpatient clinic and theatre	consultation with outpatients in clinic	PATIENT CONSULTATION	WORK ACTIVITY OF HEALTHCARE PROFESSIONALS
we see outpatients	consultation with outpatients in clinic	PATIENT CONSULTATION	WORK ACTIVITY OF HEALTHCARE PROFESSIONALS
ward rounds	consultation with inpatients in the ward	PATIENT CONSULTATION	WORK ACTIVITY OF HEALTHCARE PROFESSIONALS
trauma referral screening/Accepting	VULA enables patient referral from other hospitals	REMOTE REFERRALS	WORK ACTIVITY OF HEALTHCARE PROFESSIONALS
taking referrals from outside of the hospital	reception of referrals	REMOTE REFERRALS	WORK ACTIVITY OF HEALTHCARE PROFESSIONALS
Accept VULA requests	reception of referrals	REMOTE REFERRALS	WORK ACTIVITY OF HEALTHCARE PROFESSIONALS
our referrals come in via VULA	reception of referrals	REMOTE REFERRALS	WORK ACTIVITY OF HEALTHCARE PROFESSIONALS
Referring patients to other colleagues	VULA enables patient referral from other hospitals	REMOTE REFERRALS	WORK ACTIVITY OF HEALTHCARE PROFESSIONALS
It is the official referral route for our department	VULA enables patient referral from other hospitals	REMOTE CONSULTATION	PURPOSE OF IMPLEMENTED HITs
Decide whether or not you need to see the patient	VULA enables patient referral from other hospitals	REMOTE CONSULTATION	PURPOSE OF IMPLEMENTED HITs
ask advice	VULA enables consultation between healthcare professionals	REMOTE CONSULTATION	PURPOSE OF IMPLEMENTED HITs
Advice on treatment plan	VULA enables consultation between healthcare professionals	REMOTE CONSULTATION	PURPOSE OF IMPLEMENTED HITs
Advice on first-aid treatment before referral	VULA enables consultation between healthcare professionals	REMOTE CONSULTATION	PURPOSE OF IMPLEMENTED HITs
send picture	information exchange	FACILITATE COMMUNICATION	PURPOSE OF IMPLEMENTED HITs
Send-X-rays	information exchange	FACILITATE COMMUNICATION	PURPOSE OF IMPLEMENTED HITs
Communicating with referring colleagues	VULA facilitates remote communication between healthcare professionals	FACILITATE COMMUNICATION	PURPOSE OF IMPLEMENTED HITs
Contact with doctor on call	communication	FACILITATE COMMUNICATION	PURPOSE OF IMPLEMENTED HITs
allows direct communication with referring practitioners	VULA facilitates remote communication between healthcare professionals	FACILITATE COMMUNICATION	PURPOSE OF IMPLEMENTED HITs
the app streamlines our communication with outside centres	VULA facilitates remote communication between healthcare professionals	FACILITATE COMMUNICATION	PURPOSE OF IMPLEMENTED HITs
Teach the doctor	learning and development tool for healthcare professionals	TEACHING TOOL	PURPOSE OF IMPLEMENTED HITs
Access to X-rays	PACS enables access to patients' medical image records	FACILITATE ACCESS TO PATIENT INFORMATION	PURPOSE OF IMPLEMENTED HITs
Access to all laboratory examinations in public hospitals X2	IS/IT enables access to patient laboratory records	FACILITATE ACCESS TO PATIENT INFORMATION	PURPOSE OF IMPLEMENTED HITs
Short history	digitised records	FACILITATE ACCESS TO PATIENT INFORMATION	PURPOSE OF IMPLEMENTED HITs
Scanned documents	digitised records	FACILITATE ACCESS TO PATIENT INFORMATION	PURPOSE OF IMPLEMENTED HITs
Store X-rays	PACS enables storage of patients' medical image records	FACILITATE INFORMATION STORAGE	PURPOSE OF IMPLEMENTED HITs
keeps a record of all interaction	storage of information	FACILITATE INFORMATION STORAGE	PURPOSE OF IMPLEMENTED HITs
Database of radiology	storage of patients' information	FACILITATE INFORMATION STORAGE	PURPOSE OF IMPLEMENTED HITs
Store all patient information	storage of patients' information	FACILITATE INFORMATION STORAGE	PURPOSE OF IMPLEMENTED HITs
Requesting special investigations on patients	booking request	FACILITATE BOOKING REQUESTS	PURPOSE OF IMPLEMENTED HITs
Book X-rays on a computer system	booking request	FACILITATE BOOKING REQUESTS	PURPOSE OF IMPLEMENTED HITs
Book waiting list	booking request	FACILITATE BOOKING REQUESTS	PURPOSE OF IMPLEMENTED HITs
Admission	HITs is used to support nursing administrative duties	FACILITATE PATIENT ADMINISTRATION	PURPOSE OF IMPLEMENTED HITs
Register patients	HITs is used to support nursing administrative duties	FACILITATE PATIENT ADMINISTRATION	PURPOSE OF IMPLEMENTED HITs
Transfer patients	HITs is used to support nursing administrative duties	FACILITATE PATIENT ADMINISTRATION	PURPOSE OF IMPLEMENTED HITs
Discharged	HITs is used to support nursing administrative duties	FACILITATE PATIENT ADMINISTRATION	PURPOSE OF IMPLEMENTED HITs
Digitisation of records	HITs is used to facilitate digitisation of patient records in hospital H1	FACILITATE PATIENT ADMINISTRATION	PURPOSE OF IMPLEMENTED HITs
Take patients off system if discharged or die	HITs is used to keep track of patient treatment in hospital H1	FACILITATE PATIENT ADMINISTRATION	PURPOSE OF IMPLEMENTED HITs
Order food	HITs is used to support nursing administrative duties in hospital H1	RESOURCE MANAGEMENT	PURPOSE OF IMPLEMENTED HITs

Order stock	HITs is used to support nursing administrative duties in hospital H1	RESOURCE MANAGEMENT	PURPOSE OF IMPLEMENTED HITs
Chat application	chat tool	INTERACTION MOMENTS	PURPOSE OF IMPLEMENTED HITs
Take photos	app functionality - snap pictures	INTERACTION MOMENTS	PURPOSE OF IMPLEMENTED HITs
Name of patient	type of patient information	INTERACTION MOMENTS	PURPOSE OF IMPLEMENTED HITs
Visual acuity	VULA functionality	INTERACTION MOMENTS	PURPOSE OF IMPLEMENTED HITs
Do planning	PACS functionality	INTERACTION MOMENTS	PURPOSE OF IMPLEMENTED HITs
Draw your lines	PACS functionality	INTERACTION MOMENTS	PURPOSE OF IMPLEMENTED HITs
Do your templating	PACS functionality	INTERACTION MOMENTS	PURPOSE OF IMPLEMENTED HITs
bleep from a switchboard and then you'd have to phone switch	Bleeper facilitates communication	INTERACTION MOMENTS	PURPOSE OF IMPLEMENTED HITs
Write referral	VULA app functionality	INTERACTION MOMENTS	PURPOSE OF IMPLEMENTED HITs
See what you're dealing with	VULA enables consultation between healthcare professionals	HITs FUNCTION	PURPOSE OF IMPLEMENTED HITs
use VULA app	health ICT used	NAME OF HEALTH ICT/HIT	PURPOSE OF IMPLEMENTED HITs
Most of our work is reliant on PACS	health HIT used	NAME OF HEALTH ICT/HIT	PURPOSE OF IMPLEMENTED HITs
ECM system	health HIT used	NAME OF HEALTH ICT/HIT	PURPOSE OF IMPLEMENTED HITs
PACS	ICT used	NAME OF HEALTH ICT/HIT	PURPOSE OF IMPLEMENTED HITs
Get valuable feedback on management of patients	improved consultation between healthcare professionals	IMPROVED TREATMENT PLAN	OUTCOME OF HITs USE FOR WORK ACTIVITIES
Amount of patients accepted is much less	reduced number of patients	IMPROVED REFERRAL PROCESS	OUTCOME OF HITs USE FOR WORK ACTIVITIES
A lower number of patients to be seen at clinics	reduced number of patients	IMPROVED REFERRAL PROCESS	OUTCOME OF HITs USE FOR WORK ACTIVITIES
Lessen the burden of trauma department	reduced number of patients	IMPROVED REFERRAL PROCESS	OUTCOME OF HITs USE FOR WORK ACTIVITIES
Achieved to limit unnecessary referrals	reduced number of patients	IMPROVED REFERRAL PROCESS	OUTCOME OF HITs USE FOR WORK ACTIVITIES
referring is much more comprehensive	VULA has improved patient referral from other hospitals	IMPROVED REFERRAL PROCESS	OUTCOME OF HITs USE FOR WORK ACTIVITIES
Simplified the referral process	VULA has improved patient referral from other hospitals	IMPROVED REFERRAL PROCESS	OUTCOME OF HITs USE FOR WORK ACTIVITIES
Speeds follow-up with patient	quicker execution of work activities	IMPROVED PATIENT CONSULTATION	OUTCOME OF HITs USE FOR WORK ACTIVITIES
speed up all activities	quicker execution of work activities	IMPROVED PATIENT CONSULTATION	OUTCOME OF HITs USE FOR WORK ACTIVITIES
Don't waste time waiting	time efficient	IMPROVED PATIENT CONSULTATION	OUTCOME OF HITs USE FOR WORK ACTIVITIES
Saves time than having to take phone calls	time efficient	IMPROVED PATIENT CONSULTATION	OUTCOME OF HITs USE FOR WORK ACTIVITIES
X-rays available	PACS enabled the availability of patients' medical image records	ADEQUACY OF HITs	OUTCOME OF HITs USE FOR WORK ACTIVITIES
Helped to have it always available	PACS enabled the availability of patients' medical image records	ADEQUACY OF HITs	OUTCOME OF HITs USE FOR WORK ACTIVITIES
Can't lose the X-rays	PACS has improved the storage of electronic X-rays	ADEQUACY OF HITs	OUTCOME OF HITs USE FOR WORK ACTIVITIES

**DESCRIPTIVE CODES**	**CATEGORIES**		**sub-THEMES**	**THEMES**
when there is a problem with technology	faults with HITs	pain point	HITs UNRELIABILITY	CHALLENGES OF HITs USE
does not work properly	faults with HITs	pain point	HITs UNRELIABILITY	CHALLENGES OF HITs USE
Poor picture quality	poor picture quality	pain point	HITs UNRELIABILITY	CHALLENGES OF HITs USE
technology not always reliable	trust issues with HITs	pain point	HITs UNRELIABILITY	CHALLENGES OF HITs USE
back-up systems clumsy	clumsy back-up system	pain point	HITs UNRELIABILITY	CHALLENGES OF HITs USE
PACS iSite upgrade is slow	PACS is slow	pain point	SYSTEM DOWNTIME/SLOWNESS	CHALLENGES OF HITs USE
iSite PACS is slow	PACS is slow	pain point	SYSTEM DOWNTIME/SLOWNESS	CHALLENGES OF HITs USE
rare occurrences of ECM downtime	ECM system downtime	pain point	SYSTEM DOWNTIME/SLOWNESS	CHALLENGES OF HITs USE
when the ECM is down	ECM system downtime	pain point	SYSTEM DOWNTIME/SLOWNESS	CHALLENGES OF HITs USE
when PACS is offline	PACS downtime	pain point	SYSTEM DOWNTIME/SLOWNESS	CHALLENGES OF HITs USE
waiting period to scan paper into ECM	waiting periods for scanned patient information into ECM system	pain point	LONG WAITING PERIODS	CHALLENGES OF HITs USE
there is a waiting period for notes to be scanned in	waiting periods for scanned patient information into ECM system	pain point	LONG WAITING PERIODS	CHALLENGES OF HITs USE
Time-consuming process to get clinical notes scanned impromptu	time-consuming process	pain point	LONG WAITING PERIODS	CHALLENGES OF HITs USE
it takes so much time to type	time-consuming process	pain point	LONG WAITING PERIODS	CHALLENGES OF HITs USE
VULA referrals interferes	interruption during patient consultation	pain point	INTERRUPTION OF WORK ACTIVITIES	CHALLENGES OF HITs USE
Need to answer requests/questions	interruption during patient consultation	pain point	INTERRUPTION OF WORK ACTIVITIES	CHALLENGES OF HITs USE
time to look at and address the ICTs questions or demands	interruption during patient consultation	pain point	INTERRUPTION OF WORK ACTIVITIES	CHALLENGES OF HITs USE
you need to explain yourself a lot	interruption during patient consultation	pain point	INTERRUPTION OF WORK ACTIVITIES	CHALLENGES OF HITs USE
Find time during consultations	interruption during patient consultation	pain point	INTERRUPTION OF WORK ACTIVITIES	CHALLENGES OF HITs USE
Consultation disrupted by VULA referrals	interruption during patient consultation	pain point	INTERRUPTION OF WORK ACTIVITIES	CHALLENGES OF HITs USE
it wastes time	delay of work activities	pain point	UNINTENDED CONSEQUENCES	EFFECTS OF CHALLENGES

it causes issues and delays	delay of work activities	pain point	UNINTENDED CONSEQUENCES	EFFECTS OF CHALLENGES
slows down massively	delay of work activities	pain point	UNINTENDED CONSEQUENCES	EFFECTS OF CHALLENGES
can't go on with your work	inhibition of work activity progress	pain point	UNINTENDED CONSEQUENCES	EFFECTS OF CHALLENGES
You can't book a patient for any surgery	inhibition of work activity progress	pain point	UNINTENDED CONSEQUENCES	EFFECTS OF CHALLENGES
then you have to see the patient again	inhibition of work activity progress	pain point	UNINTENDED CONSEQUENCES	EFFECTS OF CHALLENGES
need to revert to paper based	revert to paper-based	pain point	UNINTENDED CONSEQUENCES	EFFECTS OF CHALLENGES
Increase in consultation time with patient	increased consultation time	pain point	UNINTENDED CONSEQUENCES	EFFECTS OF CHALLENGES
See lots of patients	healthcare professionals attend to lots of patients	pain point	PATIENT POPULATION SIZE	WORK ACTIVITY CHALLENGES
Reception of referral 12 hours after written	time-consuming process	pain point	TIME INEFFICIENT PROCESS	WORK ACTIVITY CHALLENGES
wait for referral to be taken to a department	time-consuming process	pain point	TIME INEFFICIENT PROCESS	WORK ACTIVITY CHALLENGES
Illegible handwritings	illegible patient information	pain point	INADEQUACY OF PAPER_BASED RESOURCES	WORK ACTIVITY CHALLENGES
Lost or misplaced	loss of paper records	pain point	INADEQUACY OF PAPER_BASED RESOURCES	WORK ACTIVITY CHALLENGES
documentation gets lost	loss of paper records	pain point	INADEQUACY OF PAPER_BASED RESOURCES	WORK ACTIVITY CHALLENGES
Unavailability of hardcopy X-rays anywhere and any time	Unavailability a single X-ray hardcopies at multiple places	pain point	INADEQUACY OF PAPER_BASED RESOURCES	WORK ACTIVITY CHALLENGES
Single individual with access to a computer	single access to HITs	pain point	INSUFFICIENT MATERIAL RESOURCES	WORK ACTIVITY CHALLENGES
more beneficial for the patient, definitely not for the workload	Increased workload	pain point	EFFECT OF WORK ACTIVITIES CHALLENGES	EFFECTS OF CHALLENGES
Late trauma referrals have significant complications	effect of late referrals	pain point	EFFECT OF WORK ACTIVITIES CHALLENGES	EFFECTS OF CHALLENGES
Reception in the hospital can often delay messages	network signal delays	pain point	EFFECT OF WORK ACTIVITIES CHALLENGES	EFFECTS OF CHALLENGES
Reception in hospital is unfortunately a provider issue	network signal delays	pain point	EFFECT OF WORK ACTIVITIES CHALLENGES	EFFECTS OF CHALLENGES
most people are quite negative about it	perception of healthcare professional	pain point	SELF CONSCIOUSNESS OF HEALTHCARE PROFESSIONALS	EFFECTS OF CHALLENGES
Feels unprofessional to attend to VULA referrals during consultation	perception of healthcare professional	pain point	SELF CONSCIOUSNESS OF HEALTHCARE PROFESSIONALS	EFFECTS OF CHALLENGES
it seems unprofessional to constantly be looking at your phone screen	perception of healthcare professional	pain point	SELF CONSCIOUSNESS OF HEALTHCARE PROFESSIONALS	EFFECTS OF CHALLENGES
Interaction is improved	Improved communication between healthcare professionals	gain point	IMPROVED COMMUNICATION AND CONSULTATION	ENHANCED WORK ACTIVITY
get valuable feedback on management of patients	improved consultation	gain point	IMPROVED COMMUNICATION AND CONSULTATION	ENHANCED WORK ACTIVITY
if you need to give feedback or medical referral	improved consultation	gain point	IMPROVED COMMUNICATION AND CONSULTATION	ENHANCED WORK ACTIVITY
Easy access to patient information from any computer at any time	Ease of access	gain point	IMPROVED ACCESS TO PATIENT INFORMATION	ENHANCED WORK ACTIVITY
Quick access	facilitate quick access	gain point	IMPROVED ACCESS TO PATIENT INFORMATION	ENHANCED WORK ACTIVITY
helped us significantly with accountability	information acquisition	gain point	ACCOUNTABILITY	ENHANCED WORK ACTIVITY
way of tracking patient	trace referral information	gain point	ACCOUNTABILITY	ENHANCED WORK ACTIVITY
it is obviously traceable	trace referral information	gain point	ACCOUNTABILITY	ENHANCED WORK ACTIVITY
enter patient information	record patient information		INFORMATION MANAGEMENT	PATIENT AND INFORMATION MANAGEMENT
write continuous report	record patient information		INFORMATION MANAGEMENT	PATIENT AND INFORMATION MANAGEMENT
register patient details	record patient information		INFORMATION MANAGEMENT	PATIENT AND INFORMATION MANAGEMENT
access to radiology done remotely	facilitate access to patient information		INFORMATION MANAGEMENT	PATIENT AND INFORMATION MANAGEMENT
notes scanned into ECM system	facilitate access to patient information		INFORMATION MANAGEMENT	PATIENT AND INFORMATION MANAGEMENT
access to electronic systems	facilitate access to patient information		INFORMATION MANAGEMENT	PATIENT AND INFORMATION MANAGEMENT
nurse can have access to enter whatever goes wrong with the patient	facilitate access to and entry of patient information		INFORMATION MANAGEMENT	PATIENT AND INFORMATION MANAGEMENT
have patient information readily	readily available patient information		INFORMATION MANAGEMENT	PATIENT AND INFORMATION MANAGEMENT
database of future reference and medico legal issues can be referenced	storage of patient information		INFORMATION MANAGEMENT	PATIENT AND INFORMATION MANAGEMENT
latest notes of a patient on phone	availability of updated patient information		INFORMATION MANAGEMENT	PATIENT AND INFORMATION MANAGEMENT
Mobile data or Wi-Fi access	Wi-Fi access		INTERNET CONNECTIVITY	ENABLING CONDITIONS FOR WORK ACTIVITIES
provide Wi-Fi that can ensure attention to referrals without the delay	Wi-Fi access		INTERNET CONNECTIVITY	ENABLING CONDITIONS FOR WORK ACTIVITIES
reliable internet connection	availability of adequate network signal		INTERNET CONNECTIVITY	ENABLING CONDITIONS FOR WORK ACTIVITIES
Faster internet	faster internet		INTERNET CONNECTIVITY	ENABLING CONDITIONS FOR WORK ACTIVITIES
